# Political economy analysis of universal health coverage and health financing reforms in low- and middle-income countries: the role of stakeholder engagement in the research process

**DOI:** 10.1186/s12961-021-00788-w

**Published:** 2021-12-11

**Authors:** Giulia Loffreda, Kéfilath Bello, Joël Arthur Kiendrébéogo, Isidore Selenou, Mohamed Ali Ag Ahmed, Jean Paul Dossou, Sophie Witter, Maria Paola Bertone

**Affiliations:** 1 Institute for Global Health and Development, Queen Margaret University, Edinburgh, UK; 2 Centre de Recherche en Reproduction Humaine et en Démographie, CERRHUD, Cotonou, Benin; 3 Department of Public Health, Institute of Tropical Medicine, Antwerp, Belgium; 4 Recherche Pour la Santé et le Développement, RESADE, Ouagadougou, Burkina Faso; 5 Heidelberg Institute of Global Health, Medical Faculty and University Hospital, Heidelberg University, Heidelberg, Germany; 6 Department of Public Health, Health Sciences Training and Research Unit, University Joseph Ki-Zerbo, Ouagadougou, Burkina Faso; 7 Research for Development International, Yaoundé, Cameroon; 8 École de Santé Publique de l’Université de Montréal, Montréal, Canada; 9 Faculty of Medicine and Odontostomatology of Bamako, Bamako, Mali

**Keywords:** Political economy, Universal health coverage, Health financing, Stakeholder engagement, Research uptake

## Abstract

**Background:**

Progress towards universal health coverage (UHC) is an inherently political process. Political economy analysis (PEA) is gaining momentum as a tool to better understand the role of the political and economic dimensions in shaping and achieving UHC in different contexts. Despite the acknowledged importance of actors and stakeholders in political economy considerations, their role in the PEA research process beyond “study subjects” as potential cocreators of knowledge and knowledge users has been overlooked so far. We therefore aimed to review the approaches with reference to stakeholder engagement during the research process adopted in the current published research on the political economy of UHC and health financing reforms, and the factors favouring (or hindering) uptake and usability of PEA work.

**Methods:**

We reviewed the literature to describe whether, when and how stakeholders were involved in the research process of studies looking at the political economy of UHC and health financing reforms, and to identify challenges and lessons learned on effective stakeholder engagement and research uptake. We used a standardized search strategy with key terms across several databases; we screened and included articles that focused on PEA and UHC. Additionally, we conducted a short survey of the authors of the included studies to complement the information retrieved.

**Results:**

Fifty articles met the inclusion criteria and were included in the analysis. We found overall little evidence of systematic engagement of stakeholders in the research process, which focused mostly on the data collection phase of the research (i.e., key informant interviews). Our study identifies some reasons for the varying stakeholder engagement. Challenges include PEA requiring specific skills, a focus on sensitive issues, and the blurriness in researchers’ and stakeholders’ roles and the multiple roles of stakeholders as research participants, study subjects and research users. Among the approaches that might favour usability of PEA work, we identified early engagement, coproduction of research questions, local partners and personal contact, political willingness, and trust and use of prospective analysis.

**Conclusions:**

Stakeholder engagement and research uptake are multifaceted concepts and complex processes, particularly when applied to PEA. As such, stakeholder engagement in the research process of PEA of UHC and health financing reforms is limited and underreported. Despite the challenges, however, stakeholder engagement remains key to ensuring relevance, usability and research uptake of PEA studies. More efforts are required to ensure engagement at different stages of the research process and better reporting in published articles.

## Introduction

### Background

Achieving universal health coverage (UHC) means ensuring access to healthcare for all as well as financial risk protection (Box 1) and is one of the targets of the Sustainable Development Goals (SDG 3.8).

Box 1: WHO’s definition of UHC UHC means that all people and communities can use the promotive, preventive, curative, rehabilitative and palliative health services they need, of sufficient quality to be effective, while also ensuring that the use of these services does not expose the user to financial hardship.

This definition of UHC embodies three related objectives:

1. Equity in access to health services—everyone who needs services should get them, not only those who can pay for them.
2. The quality of health services should be good enough to improve the health of those receiving services.
3. People should be protected against financial risk, ensuring that the cost of using services does not put people at risk of financial harm.

Source: WHO. 2019. Universal Health Coverage and Health Financing. https://www.who.int/health_financing/universal_coverage_definition/en/

It is increasingly recognized that progress towards UHC is an inherently political process, in the sense that it entails contestation over power and resources, is influenced by interests, ideas and ideologies, and as such is incremental and context-specific [1, 2]. Because of the political nature of UHC processes, political economy analysis (PEA), which brings together the economics of reforms with the politics of change, is deemed to be particularly relevant to investigate them [3].

In recent years, the empirical and theoretical literature applying PEA to UHC and health financing reforms has been growing [4–7]. This literature increasingly calls for specific awareness on the political dimensions and the economy of UHC, as well as “the imperative for the technical to engage the political—and vice versa” [8]. Much of the concluding reflections of a recent systematic review of the political economy of UHC [8] focus on methodological aspects of PEA of UHC and how/what could provide an appropriate framing to bring together political and technical analyses. In line with Sparkes et al. [9], the authors find that “there is a need to build the theoretical and practical frameworks that would enable competent and consistent analysis, the necessary funding to expand that research and the policy preparedness to engage its findings and implications” [8].

Our study builds on these reviews and propositions. However, we depart from the content and methodological focus to explore an aspect of the PEA of UHC which has been somewhat overlooked so far, that of stakeholder engagement. We noted that all frameworks and theoretical work available stress the central role of agency/actors and stakeholders [9, 10], and that existing literature proposes methods and approaches to capture their roles (such as stakeholder mapping [11] or social network analysis [12]). In parallel, there is an increasing awareness of the importance of stakeholders not only as “study subjects” but also as cocreators of knowledge and knowledge users [13, 14], in order to ensure the applicability and relevance of the research and its findings. In practice, however, it appears that knowledge coproduction might present specific challenges when applied to PEA of UHC. While we describe the challenges and identify potential approaches to address them in a companion empirical paper [15], here our aim is to explore the existing literature on the political economy of UHC to explore the role of, and approaches to, stakeholder engagement during the research process and attempt to identify lessons learned on barriers and facilitators to stakeholder engagement for PEA of UHC.

To achieve this, our review focuses on the dynamics of stakeholder engagement during research processes that use a political economy lens (i.e., PEA or other similar approaches). By “stakeholders” we mean any actor or institution with an interest or concern in the topic under study and its outcome [16], whether at national, subnational but also global level. For the case of UHC, it might include, for example, policy-makers at the ministry of health, ministry of finance, presidential office, and other relevant institutions, health managers at the district level, donors at the national or global level, implementing agencies (e.g., health insurance agencies), professional organizations or representative body (e.g., doctors’ associations, nurses’ association, relevant trade unions, and similar), private and private not-for-profit sectors (e.g., associations of private health providers, associations of faith-based providers, pharmaceutical companies), national and international nongovernmental organizations, civil society organizations and community-based organizations (CBOs)/community groups, healthcare beneficiaries or patients and lay community members. Importantly, researchers themselves are potentially included among the stakeholders. Indeed, because of their positionality in relation to the study setting, their ideologies and values and the reflexivity they exercise in relation to their work might place them in a position where they are effective stakeholders on the topic of analysis [17]. As a caveat, while we tend to refer to stakeholders as a homogeneous group for the purposes of the analysis in this study, it is important to acknowledge that the term includes a multiplicity of actors (as listed above) with differing and often conflicting ideologies, interests and power. This necessarily has an impact on the approaches to, and the outcomes of engaging with each category of stakeholder [18].

We refer to “stakeholder engagement” as the process of actively engaging actors who are involved in the UHC and health financing reforms at different stages of the research process (not only at the final stage, i.e., the “dissemination” of results). This is in line with current definitions and approaches to research uptake which stress that, to be successful, it should focus not only on the communication of the research, but also on the engagement and capacity-building of key actors, as well as the monitoring and evaluation of uptake [19]. For UHC and its PEA, stakeholder engagement is particularly important, especially to ensure research uptake of findings for effective policy-making and policy implementation [20]. Evidence suggests that there is low uptake of UHC research findings [21], which could be linked to the complex interactions among ideas, ideologies and interests of the actors involved in the UHC process [22–24]. This points towards the need to engage stakeholders at different stages of a research process to ensure that ideas and interests are clearly identified, and research framed and conducted in such a way as to increase its chances of having a policy and practice impact.

### Rationale and research objectives

The motivation for this body of research is to unpack the “so what?” question of PEA work on UHC, that refers to understanding and providing the empirical implications of the research carried out. Our starting point is the realization that, while the literature acknowledges the relevance and importance of exploring the political elements of UHC processes, one of the main challenges of PEA (and policy analysis, more broadly) for UHC and health financing reforms is that it might not always find immediate application for ongoing and future policy-making and policy implementation processes. It is often retrospective and targeted to research-oriented audiences with a methodological focus, while its uptake and usability by policy-makers and stakeholders is less explored. Stakeholder engagement, and in particular early and continuous stakeholder and research user engagement, is widely acknowledged by both researchers and funding organizations to be a central and essential element for successful research uptake [25–28]. With this study, we aimed to review the approaches with reference to stakeholder engagement during the research process adopted in the current published research on the political economy of UHC and health financing reforms, and the factors favouring (or hindering) uptake and usability of PEA work. In terms of thematic focus, we put particular attention to health financing reforms, since this element of UHC has been particularly emphasized in the literature [29]. We decided to focus our study on low- and middle-income countries (LMICs) for several reasons: Firstly, the literature on health policy and policy processes has historically focused on high-income countries (HIC); therefore, the aim of this paper is to deepen health policy analysis work in settings that may have been overlooked by this field of research [3]. With regard to UHC, and health reforms in general, LMICs express context-specific dynamics that differ from those of HICs; examples of these peculiar aspects may include a set of important political and policy actors (international actors—multilateral organizations such as the International Monetary Fund, the World Bank, WHO and the United Nations itself, bilateral donors, international nongovernmental organizations, and the more recently influential philanthropic organizations and public–private partnerships). In a context of high donor dependency, donors represent key stakeholders; donor rules shape the composition and structure of actors in the networks, which enables the entry and dissemination of new ideas and shifts in the overall balance of interest power, ultimately leading to policy change [30]. Global health actors usually have a high degree of influence on national health systems in LMICs, and the effects can be seen through different mechanisms, some with positive effects and others with negative effects. Examples of the latter could be the distortion of national health priorities [31, 32], provoking conflicts among national actors over resources and national priorities [33], spreading oversimplified solutions [34], limiting the scope of policy debate [35] and weakening national capacity when trying to regulate unhealthy commodities [36]. Finally, LMICs are generally characterized by a higher degree of fragmentation of health systems compared to HICs (e.g., pluralism with a mix of private and public sector providers; HIV/AIDS initiatives and vertical programmes).

The specific research questions guiding this review are as follows:

- What was the role of stakeholders during the different phases of the research process including (i) funding and commissioning of the research, (ii) design of the research, (iii) data collection stage, (iv) data analysis stage, (v) communication and dissemination of results, and (vi) monitoring and evaluation of research uptake?
- What are the approaches to stakeholder engagement that researchers adopted during the research process which emerge from the literature?
- Which factors/elements of these approaches favour uptake and usability of PEA work?

## Methods

### Study design

We conducted a narrative review looking at stakeholder engagement during the different phases of the research process in studies of UHC and health financing reforms, adopting a political economy framework. We adopted this study design because we considered it to be best suited to answer our research questions. However, as outlined in the PRISMA diagram in Annex 1, Fig. 4, we proceed following systematic review guidelines [37] to ensure a systematic approach to our review. We complemented information extracted from the literature reviewed with a survey sent to the authors of the included studies.

**Fig. 1:**
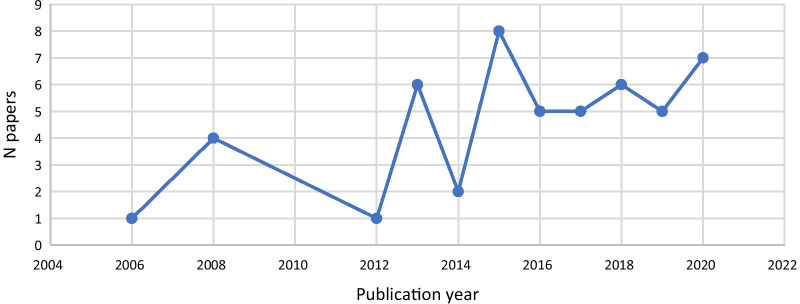
Publication years of included studies

### Search strategy

After different attempts made to optimize the literature search, we used the following search terms: (“political economy”) AND ((“universal health coverage”) OR (“health financing”)), and relevant variations adapted to the different databases (i.e., Medline/PubMed, Scopus, Google Scholar, WHO IRIS, World Bank) we searched (see Box 2). We then compared the list of articles included in Rizvi et al. to ensure that our list was as comprehensive as possible.

Box 2. Databases searched and corresponding search strategies

**Table:** 

Databases	Search strategy
PubMed	(“political economy”) AND ((“universal health coverage”) OR (“health financing”))
Google Scholar	“political economy” AND (“universal health coverage” OR “health financing”)
Scopus	{political economy} AND {universal health coverage OR health financing}
WHO IRIS	“political economy” AND “universal health coverage”
World Bank	“political economy” AND “universal health coverage”

### Study selection and inclusion criteria

In order to be included in the study, articles had to meet the following inclusion criteria:

- Focuses on concepts relating to UHC and health financing reforms, including universal coverage/universal healthcare reforms, social health protection, social health insurance, health financing reforms, health expenditure/allocation, in line with Rizvi et al. [8].
- Adopts a political economy perspective or framing, including PEA, policy analysis, policy-making analysis, policy implementation analysis, political settlements analysis, social network analysis, science–policy interface, as well as stakeholder mapping, stakeholder value network analysis, stakeholder impact analysis, in line with Rizvi et al. [8].
- Any type of empirical study, including case studies, comparative case studies, qualitative studies and mixed-methods studies, from peer-reviewed and published grey literature. However, editorials, commentaries and papers presenting theoretical frameworks were excluded.
- Setting: LMICs.
- Language restrictions: English, French and Spanish.
- Time frame: from 2005 (when universal coverage was cited in a World Health Assembly resolution [38]) to October 2020, in line with Rizvi et al. [8].

### Screening

Title and abstract screening of the retrieved articles was conducted on an Excel sheet. After removal of duplicates, titles and abstracts were first screened by two reviewers (GL, MB) based on the above selection criteria. Studies were then either included for full-text screening, excluded, or marked as “maybe”. Studies marked as “maybe” were reviewed in a second round of screening by one of the reviewers, and any disagreement was resolved by consultation.

### Data extraction

Qualitative, textual data were extracted from the documents identified into an Excel sheet, using a series of predefined themes/elements in relation to the possible approaches to/elements of stakeholder engagement at the different stages of the research process. Examples of the themes include engagement in research design, data collection and data analysis. In addition, descriptive elements of the study were also extracted to track information on, for example, topic of focus (e.g., aspect of UHC or health financing reform), country of relevance, authorship and type of study (Annex 2).

### Survey of authors of selected studies

During the data extraction process, we noted that information on the research uptake process is often not included in the published articles, due to space constraints or because it was not considered relevant for the publication. Therefore, in order to collect additional information on stakeholder engagement undertaken during the research process, we also contacted the authors of the included studies via email and asked them to fill in an online survey (developed using Microsoft Forms) with questions regarding the process of stakeholder engagement and research uptake (for the survey questions, see Annex 4). We contacted all corresponding authors, although email addresses for 12 authors were returned as not valid. Out of the remaining 39 authors successfully contacted, we received 17 responses (44%).

### Data synthesis and analysis

Basic bibliometric analysis was conducted for the documents found. The analysis of the findings adopted a narrative approach to the synthesis of the qualitative evidence. The information extracted was analysed using the themes/elements of the data extraction matrix and looking across documents [39]. This allowed identification of patterns or differences (as well as gaps) in the approaches to stakeholder engagement described in the existing literature. We also tried to understand the reasons behind the differences observed in stakeholder engagement, for example, whether they are linked to the funder, the research team, the specific topic being looked at or the findings (e.g., degree of sensitivity). For the survey component, we conducted qualitative thematic analysis, adding complementary and additional information to the data extraction matrix. Where possible, we triangulated the information provided in the survey with that included in the corresponding published article. Findings from the literature review and the authors’ survey were synthesized and are presented together in the section below.

## Results

### Characteristics of included studies

After title, abstract and full text screening, 50 studies met the inclusion criteria (PRISMA flowchart provided in Annex 3). Included studies were published between 2005 and 2020. There was a steady increase in the number of publications over the years, with a peak in 2015 (*n* = 8) (Fig. 1).

**Fig. 2:**
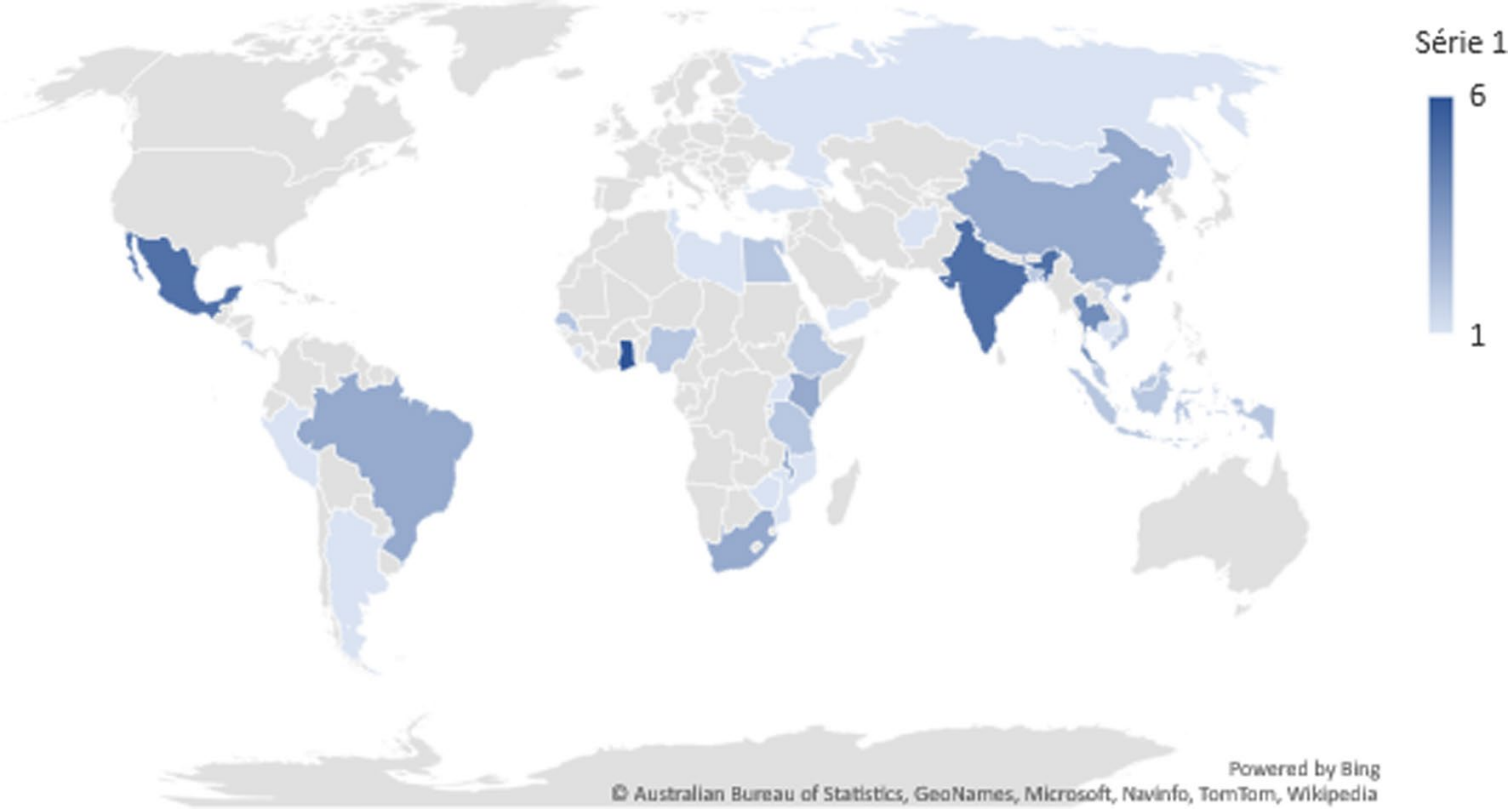
Countries under study in the included articles

The majority of studies (*n* = 36) focused on health financing reforms (e.g., social or national health insurance), and the remaining (*n* = 14) on UHC in general. As per our inclusion criteria, all included studies were in LMICs (based on the World Bank classification [40]). The largest number of studies focused on Ghana (*n* = 6), Mexico (*n* = 5), India (*n* = 5) and Thailand (*n* = 4) (Fig. 2), potentially reflecting major successful UHC reforms that have taken place in those countries. Indeed, research focus often seemed to be related to a policy or reform that had been successfully introduced or implemented, and very few papers looked at unsuccessful or failed reforms (e.g., [41]).

### Approaches to PEA and study methods

Papers included in the review adopted a variety of PEA and policy analysis approaches. While specific approaches and frameworks used differed, all aimed to understand the contextual factors and the dynamics among actors, ideas and institutions and the contestation of power and resources, with prominence given to some or all these elements. All studies included an empirical component (which was one of the inclusion criteria), so that many adopted a case study or a comparative case study design, using qualitative or mixed-methods approaches.

Most studies (*n* = 48, 94%) adopted a retrospective approach, looking back at policy processes that had happened in the past and had reached a conclusion. Only three papers carried out a prospective analysis. They used this approach to understand bottlenecks and facilitators to achieve UHC and to propose ways to improve the efficiency of health financing. One of the papers drew on a panel discussion with relevant stakeholders (e.g., Ministry of Health and Finance, WHO representatives) [42]; the other two studies aimed to revise a resource allocation formula and its implementation [43] and to predict the role played by BRICS [Brazil, Russia, India, China, South Africa] countries in the UHC movement [44].

### Stakeholder engagement in the research process

#### Researchers as stakeholders

In an attempt to reflect on the role of researchers as stakeholders, and recognizing that the design and intention of a study might depend on researchers' values, ideologies, positionality, affiliation, role, and so on, we looked at the affiliation of the authors (first author only) in relation to the study setting. Our findings show that 54% (*n* = 27) of first authors conducted the studies in a country different from their affiliation (of which 85% were affiliated with HIC institutions and studied a LMIC; the remaining were based in a LMIC institution and studied another LMIC), while for the rest of the first authors (46%, *n* = 23) the country under study corresponded to their country’s affiliation. Regarding the survey, out of 17 responses received, 11 authors were based in HIC institutions and led studies in LMICs. Five LMIC-affiliated authors conducted studies in their country of affiliation and one author in a different LMIC country. From this analysis it appears that more than a third (40%) of first authors and survey respondents conducted work in the same country as their affiliation. However, we do not have enough information on the authors’ backgrounds to fully unpack their position as stakeholders in the UHC arena. As a consequence, in the analysis below, we refer only to non-researcher stakeholders in our considerations. We further reflect on this point in the “Discussion” section.

### Research funding

Funding was clearly reported for 30 papers (58%), but it was not detailed whether the funders were part of the political economy landscape being studied. Funding for the research seems to commonly originate from external funders, such as bilateral and multilateral development agencies (World Bank, United Kingdom’s Department for International Development [DFID], WHO), foundations (Rockefeller Foundation) and research funding organizations (International Development Research Centre [IDRC], Overseas Development Institute [ODI], European Union’s Seventh Framework Programme). Only a few were funded by local research centres or universities. There is no clear evidence of whether the research funders might have influenced the research processes and findings.

### Research design

Engagement of stakeholders at the stage of research design is rarely reported in the literature we reviewed. Indeed, only one study, looking at the process of establishing the national health insurance scheme in Uganda, reported stakeholder engagement at the research design stage [45]. It was mentioned that the study was part of the work programme of the Ugandan health sector, defined and approved by the government, donors and all stakeholders, as indicated in the second National Health Policy and Health Sector Strategic and Investment plans.

However, the published literature may provide an underestimation of the stakeholders’ involvement at the design stage, as we obtained further information from the authors’ survey. Five respondents mentioned that either stakeholders were engaged in the design process by providing the conceptual framework for the study, or they initiated the research process by demanding specific evidence. In other cases, policy-makers were part of the research team and contributed to the research design, or the researchers were also policy-makers. Several authors in the studies reviewed acknowledged their role and participation in the policy reform of the country or being closely connected with the policy-making process.

### Data collection

Several papers (*n* = 22, 43%) did not include any stakeholders in the data collection process (i.e., only document review was conducted). The remaining (*n* = 29, 57%) involved stakeholders, mainly as key informants being interviewed and/or sharing relevant documentation. We could not find evidence of stakeholders who were driving or leading the process of data collection.

**Fig. 3:**
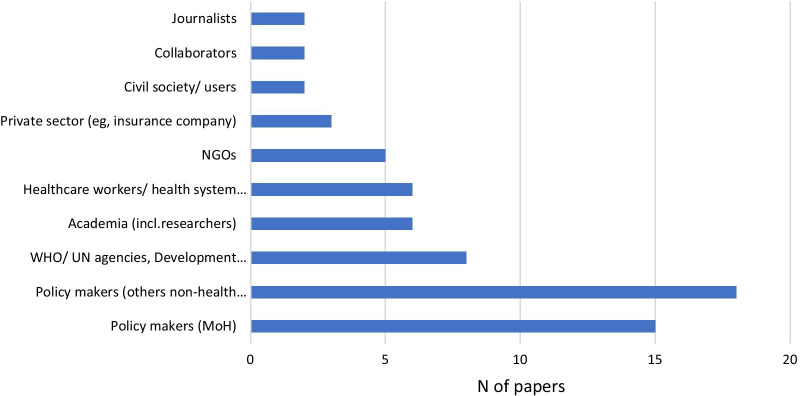
Main types of actors mentioned in the included papers that have been engaged in the data collection process

**Fig. 4:**
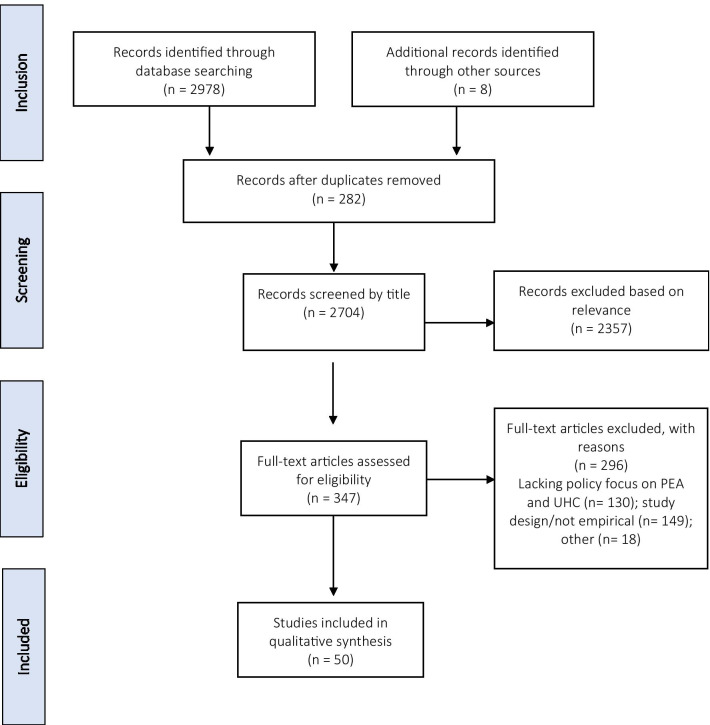
PRISMA flow diagram

The average number of stakeholders interviewed was 20. The actors involved in data collection were mostly government representatives or ministries (e.g., ministry of health, finance) and health workers/health system officials (Fig. 3). The actors least involved were civil society, researchers and the private sector, such as insurance companies.

### Data analysis

In terms of data analysis, reporting in the published literature might be somewhat underestimating the role of stakeholders for the interpretation and analysis of the data. Three papers reported the involvement of stakeholders for the validation of findings. For instance, with reference to social health insurance in Nigeria, Onoka et al. [46] mentioned that they conducted a post-study workshop where they presented the preliminary findings to study participants in order to discuss and validate the analysis and reach consensus on the results of the analysis. Similarly, Tangcharoensathien et al. [47] involved policy actors, civil society representatives and academia in the data analysis for their study in Thailand, while Musango et al. [42] based their study on a joint panel discussion with ministries, WHO Regional Office for Africa and others who participated in the meeting. In addition to this (unreported in the respective published documents), a few (*n* = 3) authors confirmed in the survey that they had shared preliminary findings with stakeholders, who were able to provide feedback.

### Knowledge translation: communication and dissemination of findings, and monitoring and evaluation of research uptake

Two studies, by Musango et al. and Twea et al., reported how findings were shared and disseminated [42, 43]. In these studies, the dissemination was part of the discussions that took part as part of the research process. However, as for the elements above, these findings are likely to reflect an underreporting, potentially due to limited space in published papers or the information not being considered a priority for academic publications. The authors’ survey brought more information to our attention. For instance, several authors (*n* = 11) reported that they disseminated results via workshops with policy-makers or health workers, via meetings (e.g., with parliament committee, municipal officers, panels) and via conferences on health financing. They also acknowledged that essential factors for successful policy uptake are early engagement with stakeholders, coproduction of research questions, and local partners establishing key linkages with stakeholders.

However, follow-up and monitoring of the usage of research findings and actual research uptake appear to be somewhat limited, even allowing for a time lag between publication and research uptake. Information based on the survey shows that some of the authors (*n* = 7) were aware that findings were being used, for example by development partners (World Bank) or local parliaments, or they mentioned that the authors themselves held a position that allowed for uptake of research findings and recommendations.

### Recommendations included in articles reviewed

Following the point above, it seems important to analyse the types of recommendations that were made in the studies reviewed—including looking into whether they were methodological or policy recommendations and whether they pointed to general lessons learned versus context-specific ones.

Almost all papers (*n* = 43, 84%) included some type of recommendations grounded in the analysis conducted. Recommendations were made on research, such as operational or methodological aspects, as well as on policy- or governance-related aspects, or both types. Policy recommendations represented the majority of those included (*n* = 35, 68%). Overall, the approach was general (rather than context-specific), referring for example to the need to strengthen political commitment and leadership, to adopt multisectoral approaches, or to improve resource allocation and integration of financing mechanisms to achieve UHC. Other recommendations, at the intersection of PEA methods and policy, underscored the importance of taking into account interests, ideologies and power of politically and economically influential groups, the contestation of ideas and consensus among social groups, and the need to consider contextual features, such as fragmentation of health systems and the implications for UHC, among others. Only a few papers (*n* = 11, 21%) included context-specific recommendations.

Recommendations were mostly aimed at policy-makers and researchers adopting PEA approaches, with only a few papers explicitly targeting donors and international organizations.

## Discussion

This review aimed to shed light on stakeholder engagement at different stages of the research process related to PEA applied to UHC and health financing reforms. We found overall little evidence of systematic engagement of stakeholders in the research process, as detailed in the published papers. Unsurprisingly, it was revealed that stakeholder engagement is better achieved for some phases of the research process (i.e., data collection, mainly through key informant interviews), while other stages entail little or no engagement (such as research design and analysis). Stakeholders have a passive role as key informants, but do not seem to drive or lead the data collection process. Knowledge translation, such as active dissemination and communication of research findings, was described in some instances, while less was done in terms of monitoring research uptake in the long term—although there is an obvious bias in reporting on these elements, maybe due to the timing of the publication, which might predate the longer research uptake process. However, additional information from the authors’ survey did not radically change the picture.

There are different potential reasons for the varying engagement at different stages of the research process. These include the fact that PEA approaches require specific skills and knowledge (especially at the design and analysis stages) that are not always available to (non-researcher) stakeholders. Additionally, it is important to recognize that PEA often discusses sensitive topics around power dynamics, agendas and interests of those same stakeholders that should be engaged in the research process [15]. Such discussions can be challenging and therefore left to the margin or the end of the research, or not included in the published document. Our analysis also reflects on the fact that researchers are stakeholders themselves because of their ideas, values and ideologies around UHC. Researchers can be affiliated to (research) institutions in the same country that is being studied and, in some cases—although we lack full information on authors’ backgrounds to assess the frequency in our review—they might be closely involved in the reform process directly. Indeed, in such cases researchers’ and policy-makers’ positions can be multifaceted and blurred in practice. The blurriness or closeness between researcher and policy-making roles and the multiple role of stakeholders (in particular, policy-makers) as research participants, study subjects and research users pose specific challenges for PEA in terms of stakeholder engagement and research uptake, which we further describe in our empirical paper [15, 48]. However, this scenario can also bring opportunities; for instance, researchers engaged in policy have access to useful and nuanced insight into the political processes, which could help to integrate different perspectives and learnings into their work. Several PEA approaches that are participatory in nature could be considered and, when possible, adopted. Despite the fact that these methods still require more theoretical and practical conceptualization, such approaches do follow the principles of coproduction in research [48–51]. In addition to these general principles, examples of specific methods, borrowed from other disciplines (such as system thinking and social sciences), that are participatory in nature and that can be perfectly suitable for conducting and complementing PEA research include participatory stakeholder mapping jointly conducted with stakeholders [52], participatory power mapping [53], group model-building [54], and mixed-methods approaches to social network analysis [55, 56].

The concept of engagement also highlights the need to differentiate between stakeholders and “rights holders”, *who* is to consider as such and *why, when,* and *how* these two positions overlap. Particularly, when discussing governance and policy processes, a social equity approach and ownership of local knowledge can help to clarify the conceptualization of who becomes a stakeholder and who a rights holder. In line with the decolonialization discourse, clarifying the positionality of researchers and other stakeholders in the (co)production of context-specific knowledge can help in making progress towards equitable partnerships and engagement [57].

In addition to these challenges in effectively engaging with stakeholders for PEA of UHC and health financing reforms research, our analysis also acknowledges the general underreporting of stakeholder engagement in the published papers, which emerges from the survey that we conducted in parallel to the literature review. There is a sense that published literature is aimed at researchers and not directly at evidence users, and the information on engagement is therefore not included. However, parallel (unreported) processes for engagement and research uptake are sometimes put in place. The low reporting of such processes might be due to journal requirements in terms of word limit or to the timing of publication compared to research uptake processes, as well as to the sense that this information is not a priority for an academic publication. There might also be the consideration that journal articles or published research reports are not the best tool for disseminating findings to non-research audiences [58], so that there is a need to adopt other approaches (such as policy briefs, meetings) [59].

Similarly, recommendations tend to be aimed at other researchers and focus on the approach itself, stressing the relevance of policy analysis and PEA approaches to understanding UHC and health financing reforms, and highlighting methodological challenges and lessons learned in conducting this type of analysis. Context-specific recommendations are rarely included, as published research usually aims for generalizability of findings rather than context specificity [60]. This focus might create a challenge for effective research uptake, and again would require separate context-specific recommendations to be disseminated through other means.

Finally, this review highlighted that research tends to focus on “successful” reforms and to be retrospective in design. While this approach is useful for understanding what has happened and to draw lessons learned from positive cases, it also means that it might be too late to inform real-time policy processes and/or provide specific guidance for failed or stalled reform processes. The focus on successful reforms may also reflect a publication bias towards positive outcomes.

### Approaches, factors and elements that might favour uptake and usability of PEA work

Our findings highlight the role of stakeholder engagement during the research process and identify some important challenges that are specific to stakeholder engagement (and research uptake) when using PEA for the analysis of UHC and health financing reforms. They also help identify specific approaches, factors and elements that might favour uptake and usability of PEA work. In particular, in line with the broader literature on the topic [38, 39], responses from the survey stress that early engagement is key for successful policy uptake, together with coproduction of research questions. They also suggest the importance of local partners to reach out to stakeholders in a meaningful and effective way. However, there is limited evidence on the broader, more structural factors that impact stakeholder engagement, such as institutional factors, health policy research approaches and policy culture, and it is difficult to draw conclusions based on our findings. Other studies have identified personal contact, political willingness and trust among the factors that contribute to effective evidence use. Capacity-building initiatives can help to address the divide between health research and policy-makers and bridge these two worlds [58].

Additionally, the literature suggests that engaging in prospective PEA to examine policy-making and implementation processes as they happen would allow research to go beyond the current focus on stakeholder consensus on technical issues and has the potential to actively engage (some) stakeholders, and create an “epistemic community” around a shared, explicit understanding of the political dynamics that promote or hinder change, and thus influence policy change [61–65].

Finally, as coproduction of knowledge, stakeholder engagement and research uptake processes become increasingly embedded and critical parts of the research process [33, 35], it is important that these are designed and reported on (including in the published articles) in a more systematic and standardized way, including and particularly for studies adopting PEA and policy analysis approaches.

## Limitations

This is the first review that focused on understanding stakeholder engagement in the research process for studies of the political economy of UHC and health financing reforms. However, there are some limitations in our methodology and findings. This paper aimed to be an exploratory review, as neither the topic nor the aim lent itself to a systematic search and identification of the literature. Consequently, we might not have retrieved all relevant papers, and we did not assess the quality of included studies. However, care was taken during the search and selection stages to keep key words and criteria as broad as feasible, and to triangulate our included articles with a recent review on the same topic [8]. In addition, the purposeful search that we carried out helped to provide a snapshot of the literature and identify general tendencies and exceptional or outlying cases that help us draw some key findings. Despite this, we acknowledge that it is likely that there are studies of PEA of UHC and health financing reforms that remain outside the public domain, for example, because they were carried out by development partners before designing a programme or because of the sensitive nature of the topic. In fact, only a few studies were funded by local bodies, which may reflect a lack of local demand and/or too sensitive a nature for local funders to tackle these topics.

In other cases, some studies of PEA of UHC and health financing reforms, including specifically the details on stakeholder engagement that we are interested in, might have been left unpublished or under-/unreported in the available literature, or published without an explicit labelling of PEA. To address this issue, we complemented the literature review with an author survey, which undoubtedly helped to add relevant information that we may have missed otherwise. However, the response rate for the survey was relatively low, making our findings less robust and complete.

## Conclusions

In recent years, there has been a growing awareness of the importance and relevance of political economy factors that influence policy-making and implementation processes for UHC and health financing reforms. This has led to increased attention to the study and the analysis of those factors, including adopting PEA and related approaches. However, while PEA offers analysis that is compelling to explain the politics of reforms, some questions remain open in terms of the policy relevance and usability of the lessons learned from PEA, considering ongoing and future reform processes and the need to inform actions or interventions ex ante. While stakeholder engagement is unlikely to be the only step or action required to ensure research uptake of findings, it is recognized as a critical one.

In this study, we reviewed the literature to describe whether, when and how stakeholders were involved in the research process for PEA of UHC and health financing reforms, and attempted to identify challenges and summarize the key learnings on what works and what could work for stakeholder engagement and research uptake in this area. Our analysis shows that, while stakeholder engagement and research uptake are always multifaceted concepts and complex processes, they are even more so when applied to PEA. In a companion empirical paper, we explore these issues from an empirical perspective based on our own experience of engaging stakeholders in a project involving PEA of UHC, and attempt to chart a way forward.

## Data Availability

The datasets used and/or analysed during the current study are available from the corresponding author on reasonable request.

## Annex 1

See Fig. 4.

## Annex 2

See Table 1.

**Table 1:** Data extraction framework

Main category	Subcategory	Description/notes/hypotheses
Bibliometric data		
Author		Last name, first name
Author affiliation		
Title		
Link/DOI		
Year of publication		
Year study conducted		(if available)
Time frame under study		Years of studies included
Country/ies of focus		
Setting definition	HIC MIC LIC	
Study method classification	Qualitative Mixed-methods Document analysis	Primary vs secondary studies
Study sample		
Objectives/purpose		Stated objectives of the study
Policy/reform of focus		
Data on stakeholder engagement at key stages of the research process		
Funding	Who commissioned and/or funded the PEA?	
Design	Were stakeholders involved in the design of the study and definition of the research questions? If yes, who/which stakeholders? How were they involved?	
	Is the study a retrospective or prospective design?	
	What is the envisaged “audience” of the research?	
Data collection	Were stakeholders involved in data collection? If yes, who/which stakeholders, and how were they were involved?	For example (who), were stakeholders from other sectors, or non-state stakeholders involved? For example (how), as key informants, or to provide key documentation, etc.
Data analysis	Were stakeholders involved in data analysis? If yes, who/which stakeholders, and how were they were involved?	For example, brainstorming, feedback of preliminary data and/or “respondent validation” of results with key informants
Communication/dissemination of findings	How were findings shared and with whom?	Hyp. is that often findings are not disseminated locally, as seen as sensitive
	Did the study include policy-related recommendations for local change?	Versus methodological reflections or recommendations for donors or external actors
Monitoring and evaluation of research uptake	Did the study track any uptake or impact of their work/findings?	

## Annex 3

See Table 2.

**Table 2:** List of included studies

Study ID	First author	Year	Title	Country of study	Policy/reform of focus
1	Michael R Reich	2015	Moving towards universal health coverage: lessons from 11 country studies	Bangladesh, Brazil, Ethiopia, France, Ghana, Indonesia, Japan, Peru, Thailand, Turkey, and Vietnam	National Health Insurance Scheme (Ghana); service exchange agreements (Peru); tax revenues and earn marking
2	Tessa Oraro-Lawrence	2020	Policy levers and priority-setting in universal health coverage: a qualitative analysis of healthcare financing agenda setting in Kenya	Kenya	Health Financing Strategy 2016–2030, National Hospital Insurance Fund (NHIF)
3	Pakwanja Twea	2020	Allocating resources to support universal health coverage: policy processes and implementation in Malawi	Malawi	Essential Health Package (EHP)
4	Elisabeth Paul	2020	An assessment of the core capacities of the Senegalese health system to de-liver Universal Health Coverage	Senegal	Plan National de Développement Sanitaire et Social (PNDSS)2019–2028
5	Diane McIntyre	2008	Beyond fragmentation and towards universal coverage: insights from Ghana, South Africa and the United Republic of Tanzania	Ghana, South Africa and the United Republic of Tanzania	Prepayment funding mechanisms, community-based health insurance (CBHI), private voluntary health insurance
6	Fabrizio Tediosi	2015	BRICS countries and the global movement for universal health coverage	BRICS countries: Brazil, Russia, India, China and South Africa	UHC agenda in general
7	Saudamini Vishwanath Dabak	2018	Budgeting for a billion: applying health technology assessment (HTA) for universal health coverage in India	India	Health technology assessment
8	Meghan Bruce Kumar	2020	How do decision-makers use evidence in community health policy and financing decisions? A qualitative study and conceptual framework in four African countries	Ethiopia, Kenya, Malawi, Mozambique	Health financing
9	Elizabeth Pisani	2017	Indonesia's road to universal health coverage: a political journey	Indonesia	Health insurance
10	Robert K Basaza	2013	Players and processes behind the national health insurance scheme: a case study of Uganda	Uganda	Health financing
11	Bertone M	2018	The bumpy trajectory of performance-based financing for healthcare in Sierra Leone: agency, structure and frames shaping the policy process	Sierra Leone	Performance-based financing (PBF)
12	Bui TT Ha	2014	Policy processes underpinning universal health insurance in Vietnam	Vietnam	Health insurance
13	Octavio Gomez Dantes	2015	Political Economy of Pursuing the Expansion of Social Protection in Health in Mexico	Mexico	Social protection in health (SPH)
14	José Carlos R. Pueblita	2013	Screening Seguro Popular: The Political Economy of Universal Health Coverage in Mexico	Mexico	System of Social Protection in Health (SSPH)
15	Adam Fusheini	2017	Stakeholders Perspectives on the Success Drivers in Ghana’s National Health Insurance Scheme—Identifying Policy Translation Issues	Ghana	National Health Insurance Scheme (NHIS)
16	Rebecca Surender	2015	The drive for universal healthcare in South Africa: views from private general practitioners	South Africa	National Health Insurance
17	Kevin Croke	2019	The political economy of health financing reform in Malaysia	Malaysia	Health financing reforms
18	Witter S	2019	The political economy of results-based financing: the experience of the health system in Zimbabwe	Zimbabwe	Result-based financing
19	Tangcharoensathien, Viroj	2019	The Political Economy of UHC Reform in Thailand: Lessons for Low- and Middle-Income Countries	Thailand	Universal Coverage Scheme (UCS)
20	Benjamin Chemouni	2016	The political path to universal health coverage: Power, ideas and community-based health insurance in Rwanda	Rwanda	Community-based health insurance (CBHI)
21	Adam Fusheini	2016	The Politico-Economic Challenges of Ghana’s National Health Insurance Scheme Implementation	Ghana	National Health Insurance Scheme
22	Ariel Higgins-Steele	2018	Towards universal health coverage and sustainable financing in Afghanistan: progress and challenges	Afghanistan	UHC
23	Clarke, L	2017	Shocks, stresses and universal health coverage: Pathways to address resilience and health	LMIC	UHC
24	Kelsall, T	2016	Political settlements and pathways to universal health coverage	LMIC	UHC
25	Krajewski-Siuda	2008	Political analysis of the conception of the Polish National Health Fund	Poland	National health fund
26	Musango, L	2012	Moving from ideas to action—developing health financing systems towards universal coverage in Africa	African countries	Health financing
27	Chima A Onoka	2013	Promoting universal financial protection: constraints and enabling factors in scaling-up coverage with social health insurance in Nigeria	Nigeria	Formal Sector Social Health Insurance Programme
28	Vargas, JR	2013	Promoting universal financial protection: a policy analysis of universal health coverage in Costa Rica (1940–2000)	Costa Rica	UHC
29	Dennis Raphael	2020	Conceptualizing and researching health equity in Africa through a political economy of health lens—Rwanda in perspective	Rwanda	Pro-health equity policies
30	Philipa Mladovsky	2020	Fragmentation by design: Universal health coverage policies as governmentality in Senegal	Senegal	UHC (user fee exemption, reforming health insurance institutions that cover workers and pensioners in the civil service and private sector, expanding coverage of community-based health insurance [CBHI] schemes)
31	Rami Yassoub	2017	The Path Toward Universal Health Coverage: Stakeholder Acceptability of a Primary Care Health Benefits Package in Lebanon	Lebanon	Healthcare benefits package (HBP)
32	Shadi S Saleh	2014	The path towards universal health coverage in the Arab uprising countries Tunisia, Egypt, Libya, and Yemen	Tunisia, Egypt, Libya, and Yemen	UHC
33	Tim Kelsall	2016	Inclusive healthcare and the political settlement in Cambodia	Cambodia	Health Equity Fund
34	Magdalena Chiara	2018	Concepts and ideas concerning universal health care: results of the intergovernmental arrangement in Greater Metropolitan Buenos Aires, Argentina, from 2003 to 2011	Argentina	Plan Nacer and Programa Remediar/Universal coverage
35	Bernardo Garza	2015	Increasing the Responsiveness of Health Services in Mexico's Seguro Popular: Three Policy Proposals for Voice and Power	Mexico	Public insurance (social security insurance)/seguro popular
36	Sharif A Ismail	2018	The rocky road to universal health coverage in Egypt: A political economy of health insurance reform from 2005–15	Egypt	Health insurance coverage
37	Sparks, S	2019	Political Economy Analysis for Health Financing Reform	Global/Mexico/Turkey	Health financing reforms
38	Bayarsaikhan	2015	Social health insurance development in Mongolia: Opportunities and challenges in moving towards Universal Health Coverage	Mongolia	Health insurance and public financing reform
39	Frenk, J	2006	Comprehensive reform to improve health system performance in Mexico	Mexico	Seguro Popular (popular health insurance)
40	Leng, C	2008	Ownership, control, and contention: Challenges for the future of healthcare in Malaysia	Malaysia	Health financing
41	Massuda, A	2018	The Brazilian health system at crossroads: progress, crisis and resilience	Brazil	Health financing and healthcare coverage
42	Yu	2015	Universal health insurance coverage for 1.3 billion people: What accounts for China’s success?	China	UHC/UH insurance coverage
43	McIntyre, D	2013	Promoting universal financial protection: evidence from seven low- and middle-income countries on factors facilitating or hindering progress	Costa Rica, Georgia, India, Malawi, Nigeria, Tanzania, and Thailand	Health financing/health insurance
44	He AJ	2017	Towards Universal Health Coverage via Social Health Insurance in China: Systemic Fragmentation, Reform Imperatives, and Policy Alternatives	China	Universal insurance coverage
45	Viroj Tangcharoensathien	2013	Promoting universal financial protection: how the Thai universal coverage scheme was designed to ensure equity	Thailand	Thai Universal Coverage Scheme (UCS)
46	Dayashankar	2019	Policy entrepreneurs as catalysts of broad system change: the case of social health insurance adoption in India	India	Social health insurance
47	Agyepong IA	2008	Public social policy development and implementation: a case study of the Ghana National Health Insurance scheme	Ghana	National health insurance scheme
48	Shroff Z	2015	Health Insurance as a Tool of Electoral Tactical Redistribution in Tamil Nadu, India	India	National Health Insurance Scheme/social welfare programmes
49	Ishmael Wireko	2016	Transnational actors and health care reform: Why international organizations initially opposed, and later supported, social health insurance in Ghana	Ghana	NHIS
50	Osman	2018	Political economy and quality of primary health service in rural Bangladesh and the united states of America: a comparative analysis	Bangladesh	Primary healthcare (Bangladesh only)

## Annex 4. Survey to authors on the process of stakeholder engagement

(i) What was the role of stakeholders during the different phases of the research process? Please specify any of the below (and provide details, if you wish):
  - Funding/commissioning of the research
  - Design of the research and/or identification of the research question(s)
  - Data collection stage (for example, stakeholders were interviewed and/or contributed to documents to be reviewed)
  - Data analysis stage (for example, collaborative analysis, review/validation of early findings, etc.)
  - Communication/dissemination of results (for example, workshop or meetings to present and discuss results)
  - Monitoring and evaluation of research uptake (for example, feedback on use of research findings and recommendations).
(ii) Please provide details for those phases that you selected above.
(iii) Following the publication of this paper, have you conducted specific activities to facilitate the uptake of the findings? If yes, what type of activities and what type of stakeholders did you engage? Do you know what was the outcome of those research uptake activities (e.g., in terms of direct/indirect influence on policy and practice)?
(iv) Did you make any specific recommendations to the stakeholders that are specific to the setting of the study and are not included in the study itself?
(v) Are you aware whether any of the recommendations or the findings of your study have been considered and/or used by stakeholders to shape policy and/or practice?
(vi) Based on your experience with this work, what do you think are the factors and approaches to stakeholder engagement that might favour uptake and usability of your PEA? Or conversely, the factors that hampered uptake and usability of your PEA or made its use challenging?

## Abbreviations

BRICS: Brazil, Russia, India, China, South Africa
CBOs: Community-based organizations
DFID: Department for International Development
EU: European Union
IDRC: International Development Research Centre
LMICs: Low- and middle-income countries
ODI: Overseas Development Institute
PEA: Political economy analysis
UHC: Universal health coverage
